# Read clouds uncover variation in complex regions of the human genome

**DOI:** 10.1101/gr.191189.115

**Published:** 2015-10

**Authors:** Alex Bishara, Yuling Liu, Ziming Weng, Dorna Kashef-Haghighi, Daniel E. Newburger, Robert West, Arend Sidow, Serafim Batzoglou

**Affiliations:** 1 Department of Computer Science, Stanford University, Stanford, California 94305, USA;; 2 Department of Chemistry, Stanford University, Stanford, California 94305, USA;; 3 Department of Pathology, Stanford University School of Medicine, Stanford, California 94305, USA;; 4 Biomedical Informatics Training Program, Stanford, California 94305, USA;; 5 Department of Genetics, Stanford University School of Medicine, Stanford, California 94305, USA

## Abstract

Although an increasing amount of human genetic variation is being identified and recorded, determining variants within repeated sequences of the human genome remains a challenge. Most population and genome-wide association studies have therefore been unable to consider variation in these regions. Core to the problem is the lack of a sequencing technology that produces reads with sufficient length and accuracy to enable unique mapping. Here, we present a novel methodology of using read clouds, obtained by accurate short-read sequencing of DNA derived from long fragment libraries, to confidently align short reads within repeat regions and enable accurate variant discovery. Our novel algorithm, Random Field Aligner (RFA), captures the relationships among the short reads governed by the long read process via a Markov Random Field. We utilized a modified version of the Illumina TruSeq synthetic long-read protocol, which yielded shallow-sequenced read clouds. We test RFA through extensive simulations and apply it to discover variants on the NA12878 human sample, for which shallow TruSeq read cloud sequencing data are available, and on an invasive breast carcinoma genome that we sequenced using the same method. We demonstrate that RFA facilitates accurate recovery of variation in 155 Mb of the human genome, including 94% of 67 Mb of segmental duplication sequence and 96% of 11 Mb of transcribed sequence, that are currently hidden from short-read technologies.

Although next-generation sequencing (NGS) technologies have enabled whole-genome sequencing (WGS) of many individuals to identify variation, current large-scale and cost-effective resequencing platforms produce reads of limited length (Shendure and Ji 2008; Metzker 2010); and as a result, variant identification within repeated sequences remains challenging. The 1000 Genomes Project Consortium has reported that nearly 6% of the GRCh37 human genome reference is inaccessible by short-read technologies (The 1000 Genomes Project Consortium 2012). Further studies have shown that as much as 10% of GRCh37 cannot be aligned to for the purpose of accurate variant discovery (Lee and Schatz 2012).

The portion of the human genome that is currently dark to short-read technologies is significant in both its size and phenotypic effect. Recent segmental duplications (also referred to as low copy repeats), consisting of regions >5 kbp in size and >94% sequence identity, have been identified as making up 130.5 Mb, or ∼4.35% of the human genome (Bailey et al. 2002). These regions tend to be hotspots of structural and copy number variants (CNVs) (Coe et al. 2014; Chaisson et al. 2015) that in aggregate affect a larger fraction of the genome than that affected by single nucleotide polymorphisms (SNPs) (Conrad et al. 2010). CNVs have been associated with diseases such as autism (Sebat et al. 2007; Pinto et al. 2010), Crohn's disease (Wellcome Trust Case Control Consortium et al. 2010), schizophrenia (Stefansson et al. 2008; McCarthy et al. 2009), and neurocognitive disorders (Coe et al. 2014). However, current short-read technologies are unable to identify precise nucleotide variation in these regions.

In principle, longer sequencing reads provide an opportunity to disambiguate repeated sequences. Technologies such as Pacific Biosciences (PacBio) (McCarthy 2010) and Oxford Nanopore (Ashton et al. 2014) produce long reads, but at much higher per-base error rate. PacBio has been leveraged for improved bacterial reference genome assemblies (Koren et al. 2013) and for targeted de novo assembly of the complex 1.3 Mb of 17q21.31 (Huddleston et al. 2014). However, these technologies are currently substantially lower in throughput and higher in cost than short-read technologies and so cannot currently be used to cost-effectively uncover variation in repeated regions of the genome.

An alternative approach used in LFR (Peters et al. 2012), CPT-seq (Amini et al. 2014), and Illumina TruSeq Synthetic Long-Reads (previously known as Moleculo) (Kuleshov et al. 2014) utilizes accurate short-read sequencing of long DNA fragments in order to obtain long-range information at high nucleotide accuracy. The Illumina TruSeq protocol is able to produce 10-kbp long reads, retaining the benefits of the highly accurate and cost-effective Illumina technology (Kuleshov et al. 2014) and enabling human genome phasing (Kuleshov et al. 2014) and de novo assembly of complex genomes (Voskoboynik et al. 2013; McCoy et al. 2014).

Under Illumina's synthetic long-read protocol, DNA sequencing libraries are prepared as follows: First, the genomic DNA is sheared into long (≥10 kbp) fragments and ligated with amplification adapters at both ends; second, these molecules are diluted into wells so that each well receives only a small fraction (1%–2%) of the genome; third, molecules are amplified, sheared into short fragments, and uniquely barcoded within each well (Kuleshov et al. 2014). The individual wells are then pooled and sequenced together. Demultiplexing the resulting reads by well barcode and aligning them to the reference genome yields clusters of short reads, which we call *read clouds*, each of which originated from a single long DNA molecule (Fig. 1A). Additionally, short reads that originate from the endpoints of a read cloud will overlap the original adapters ligated to the long molecules and serve as end-markers of the original long molecule.

**Figure 1.**
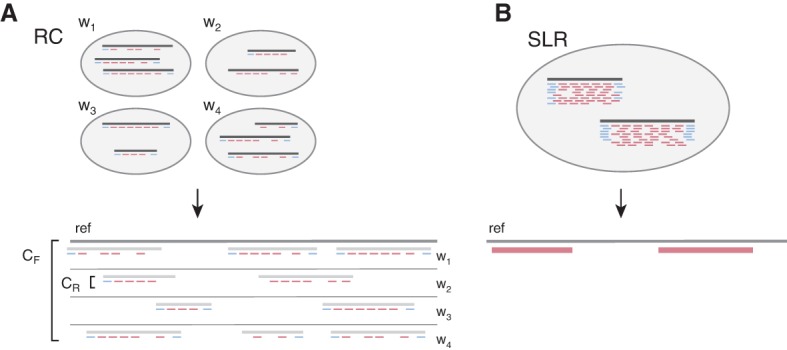
Read clouds (RC) and synthetic long reads (SLR) obtained by Illumina TruSeq Synthetic Long-Read sequencing*.* Each well initially contains long molecules that represent a small fraction of the target genome; reads from each long molecule are separated in genomic coordinates within the target genome, and therefore, clusters of such reads (read clouds) are formed with each cluster originating from one source fragment. Blue reads denote end-markers of the source fragments and may not always be present as sequenced short reads. (*A*) In the RC approach, long fragments from several wells *w*_n_ are sequenced to a shallow depth and aligned to the reference to obtain read clouds. Pooling of reads across several read clouds allows inference of the variation in the underlying long fragments. (*B*) In the SLR approach, long fragments are sequenced to a much higher depth to enable de novo assembly of synthetic long reads. For the same total sequencing budget *C*, the RC approach covers proportionally more target genome space than the SLR approach.

A read cloud approach has two key parameters for genome coverage (Fig. 1): coverage of the genome with long DNA fragments, *C*_F_, and coverage of each long fragment with short reads, *C*_R_. The total sequencing depth is then *C* = *C*_F_ × *C*_R_. The choice of *C*_F_ and *C*_R_ for a given short-read sequencing budget *C* heavily influences the ability of the read cloud approach to accurately discover variation within a target genome. Both *C*_F_ and *C* have to be sufficiently high; in particular, *C*_F_ has to be high enough so that both haplotypes of a diploid genome are covered with a sufficient number of long fragments (Lander and Waterman 1988). The original protocol (McCoy et al. 2014) required for each well to be sequenced at a high depth (*C*_R_ = 50×) in order to first de novo assemble synthetic long reads (SLR) of the original source long fragments (Fig. 1B; Voskoboynik et al. 2013). However, performing WGS with this approach requires an exorbitant amount of total sequencing in order to obtain a sufficiently high *C*_F_. For example, if *C*_R_ = 50× (Voskoboynik et al. 2013) and *C*_F_ = 20×, *C* = 50 × 20 = 1000×, or the equivalent of 33 whole human genomes sequenced at the currently standard 30× coverage.

The alternative strategy to true SLR approaches is to bypass the requirement for actual assembly of the original long fragments and to minimize short-read coverage (*C*_R_ ≤ 2×). This strategy allows a sufficiently high *C*_F_ in order to cover a genome at a reasonable coverage budget *C*. Choosing *C*_R_ = 1.5 and *C*_F_ = 20×, *C* = 1.5 × 20 = 30×, would yield valuable long-range information for the same total sequencing cost as the currently standard short-read WGS approach.

In this work, we present RFA (Random Field Aligner), a novel methodology that utilizes the high *C*_F_, low *C*_R_ read cloud approach to confidently map short reads within repetitive regions. In RFA, we directly model the short-read generative process from source long molecules in order to capture the dependencies of short reads through the hidden source long molecules. Using this probabilistic approach, we reduce the problem of finding optimal short-read alignments to optimizing a Markov Random Field (MRF). The resulting alignments tend to cluster the mapped reads into read clouds that fit the properties of the synthetic long-read sequencing protocol. The model naturally favors alignment of a read cloud to the specific copy of a repeated sequence that minimizes the sequence variation of the read cloud to the copy.

To our knowledge, RFA is the first attempt to take advantage of the long-range information present in shallow read cloud sequencing to improve the resulting short-read alignments and also to use read clouds to directly genotype an individual. Prior implementations of read clouds to provide molecular-phased genotypes for a single individual require known genotypes as input (Kitzman et al. 2011; Amini et al. 2014; Kuleshov et al. 2014) and typically align the resulting read clouds with standard short-read aligners in order to observe the allele at a known SNV within each read cloud. As genotypes are typically determined with a standard whole-genome 30× shotgun sequencing, in which a short-read workflow would be used, variants in complex regions would remain unresolved.

We demonstrate the utility of our approach using shallow-sequenced read clouds (*C*_R_ = 1.5×) obtained from the Illumina TruSeq synthetic long-read protocol (henceforth referred to as TruSeq read clouds to avoid confusion with the Illumina product that uses deep sequencing to assemble synthetic long reads). We tested our approach on simulated read cloud wells, on TruSeq read cloud libraries for the cell line GM12878 for which assembled synthetic long reads are also available for direct validation (Genomes Moleculo NA12878, 2014, ftp://ftp.1000genomes.ebi.ac.uk/vol1/ftp/phase3/integrated_sv_map/supporting/NA12878/moleculo/), and on a high coverage cancer sample that we sequenced. Evaluation of the results confirmed that our method accurately recovers precise nucleotide variation within a significant fraction of the human genome that was previously dark to current short-read technologies. We are able to leverage the read cloud strategy to recover this variation at a fraction of the cost of the original protocol and eliminate the need for first assembling synthetic long reads.

## Results

### Overview of algorithms

In order to accurately align short reads resulting from a synthetic long-read protocol, we developed a probabilistic framework to model the process by which read clouds are generated. In this framework, each well contains a set of hidden source long fragments, ***M***, that generate the short-read fragments, ***R***. Read alignment is then the problem of jointly aligning all the reads of a given well to the target genome to maximize the probability of the observed reads, *P*(***R***). The probability distribution over the reads in each well can be written as

$$P(R)=\;\underset{M}{\sum }P(R|M)P(M),$$

where *P*(***R***|***M***) is the short-read generative process from long fragments; *P*(***M***) is our prior belief over possible hidden long fragment configurations; and the sum is over all possible hidden configurations. To make computation of *P*(***R***) tractable, we developed a heuristic to first determine the candidate long fragments in each well, and with these seeds, construct a Markov Random Field (MRF) in which each candidate long molecule induces a single potential function over the reads (see Methods).

**Figure 2.**
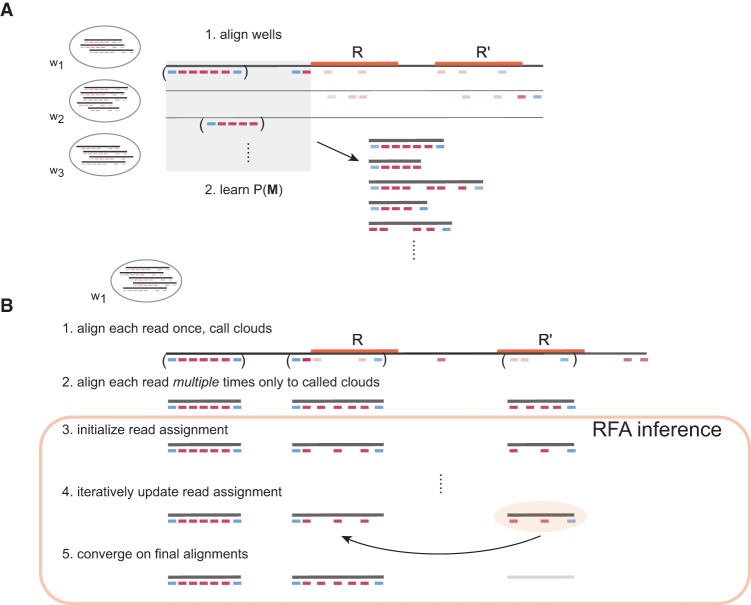
RFA overview. (*A*) Wells *w*_n_ from the sample are first aligned to the reference using an existing short-read aligner, and uniquely mapped read clouds are used to learn a prior *P*(***M***), which captures protocol properties such as the long fragment size distribution. (*B*) Each well is aligned separately with the aid of a short-read aligner to determine candidate source long fragment locations as well as multiple candidate short-read alignments to the long fragments. Finally, MAP inference is performed to converge on optimal alignments. In this example, RFA successfully determines the correct repeat copy R that overlaps with a source long fragment.

RFA leverages our model of the read cloud generation process to produce unique alignments to specific copies of repeated sequences (Fig. 2). Wells from the sample are first aligned to the reference using an existing short-read aligner. The features of uniquely mapped read clouds are used to learn *P*(***M***), which captures protocol properties such as the long fragment size distribution. Each well is then aligned separately using the following steps: First, we use an existing short-read aligner to produce multiple candidate alignments for short reads and to determine the positions of potentially sampled long fragments ***M***; second, we perform approximate inference on our model to identify a maximum a posteriori (MAP) assignment

(1) $$r^{\mathrm{MAP}}=\arg\underset{r}{\max}\;\underset{M}{\sum }P(R=r|M)P(M).$$

Last, we use this MAP assignment to compute probability queries for both short-read alignment confidence and long fragment mappability. Simulations indicated this approach to be highly accurate and efficient in contrast to sampling approaches to compute the marginal, *P*(*R*_n_), for each read. The precise definition of our framework, together with details for efficient identification of the MAP assignment ***r***^MAP^ in Equation 1 and computation of the queries, is described in Methods.

### Alignment accuracy in simulations

In order to determine the utility of RFA over the standard short-read alignment approach, we simulated read clouds from synthetic long read data as described in Methods. From these data, we used the following four different sets of alignments and quality scores that serve as comparison points:

- *Baseline* represents the standard method of aligning short-reads without the benefit of information from a long read process.
- *Naive* represents the naive approach of first creating an abbreviated reference corresponding to candidate long molecules within a well and then realigning the short reads directly to this reference.
- *RFA* is our method that utilizes the same abbreviated reference as in *naive*, and subsequently uses probabilistic inference over an MRF in order to realign the short reads to this reference.
- *Oracle* represents the theoretical upper limit of first aligning the reads to the abbreviated reference using the short-read aligner while allowing for multiple mappings, and then picking the true mapping for each read, if that mapping was returned by the short-read aligner.

**Figure 3.**
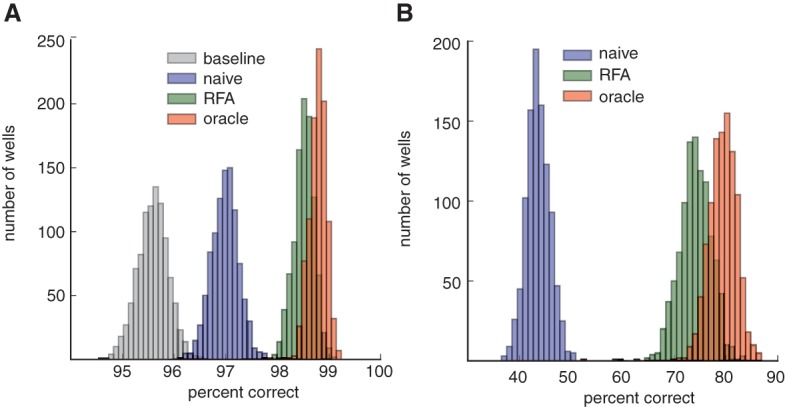
Histograms of simulation results across 1000 wells. Each point in the histogram represents the result of a single simulated well. (*A*) All reads. (*B*) Only reads that were multimapped in the abbreviated reference. RFA confidently maps an additional 2.9% (out of 3.2% from Oracle) of the total reads over the Baseline approach, and achieves 92% of the Oracle performance.

We simulated alignment of 1000 wells and computed results of each of the preceding approaches after we filtered reads with a MAPQ less than 10 (corresponding to <90% mapping confidence). The percentage of reads in each well correctly placed by RFA is very similar to Oracle, whereas the Baseline and Naive approaches have significantly lower accuracy (Fig. 3A). RFA confidently maps an additional 2.9% (out of 3.2% from Oracle) of the total reads over the Baseline approach. The results are similar when restricted to reads that were multimapped in the abbreviated reference, which signifies the usefulness of RFA over the naive approach (Fig. 3B). Our probabilistic approach with RFA is able to achieve 92% of the Oracle performance and place an additional 29% of multimapped reads confidently over the naive approach. RFA's error rate, computed by assessing the number of incorrectly placed multimapped reads with >90% confidence, was ∼1% across all simulated wells.

### Characterization of recovered regions

Our approach accurately aligns short reads to 90.6% (155 Mb) of the 171 Mb of the human genome deemed inaccessible to short-read technologies by the 1000 Genomes Project Consortium. To understand the nature of these recovered regions, we tabulated their RepeatMasker (Smit et al. 1996) and segmental duplication annotations (Bailey et al. 2001; Kent et al. 2002). We are able to recover the majority of previously ambiguous elements across all repeat categories (90%), including 94% of 67 Mb of the segmental duplications and 96% of 11 Mb of transcribed sequences that fall within the 171 Mb of currently inaccessible sequence (Table 1). Both high copy repetitive elements as well as long stretches of ambiguous segmental duplications with high sequence identity are accurately illuminated by our approach.

**Table 1.**
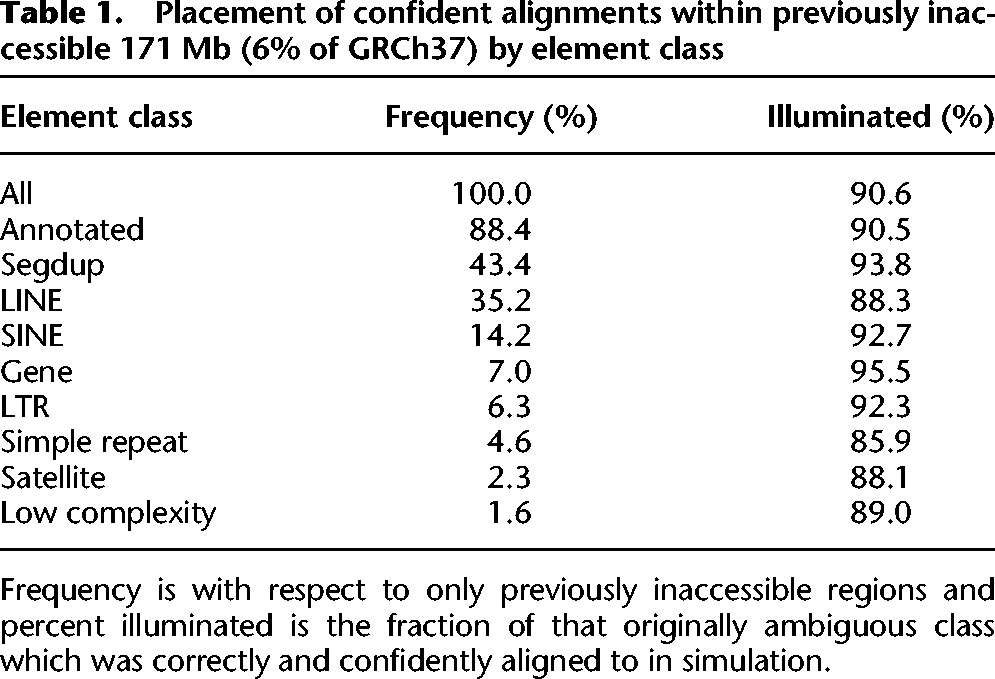
Placement of confident alignments within previously inaccessible 171 Mb (6% of GRCh37) by element class

To quantify gene content in the 155 Mb of inaccessible regions that we recovered with RFA, we used annotations from GENCODE (Harrow et al. 2012). We found that these regions cover >80% of the length of 2740 genes, and >50% of the length of 4510 genes, with both significant enrichment and depletion by type (Table 2). Examining these genes by family also shows significant enrichment and depletion (Table 3). A previous study that used copy number genotyping to examine gene families falling into paralogous regions (Sudmant et al. 2010) found several families to be highly variable and dynamic between individuals and populations. Of the highly dynamic gene families previously identified, we found *ANKRD, ZNF*, endogenous ligands*, CD*, *RBM*, and *RNF* families to be enriched in our recovered gene set. Preliminary analysis indicates that the gene content in these regions is both highly variable and also heavily understudied due to the limitations of existing NGS technologies. The nucleotide-level population variation that our method uncovers could facilitate functional annotation and disease association in these gene-rich repeat regions.

**Table 2.**
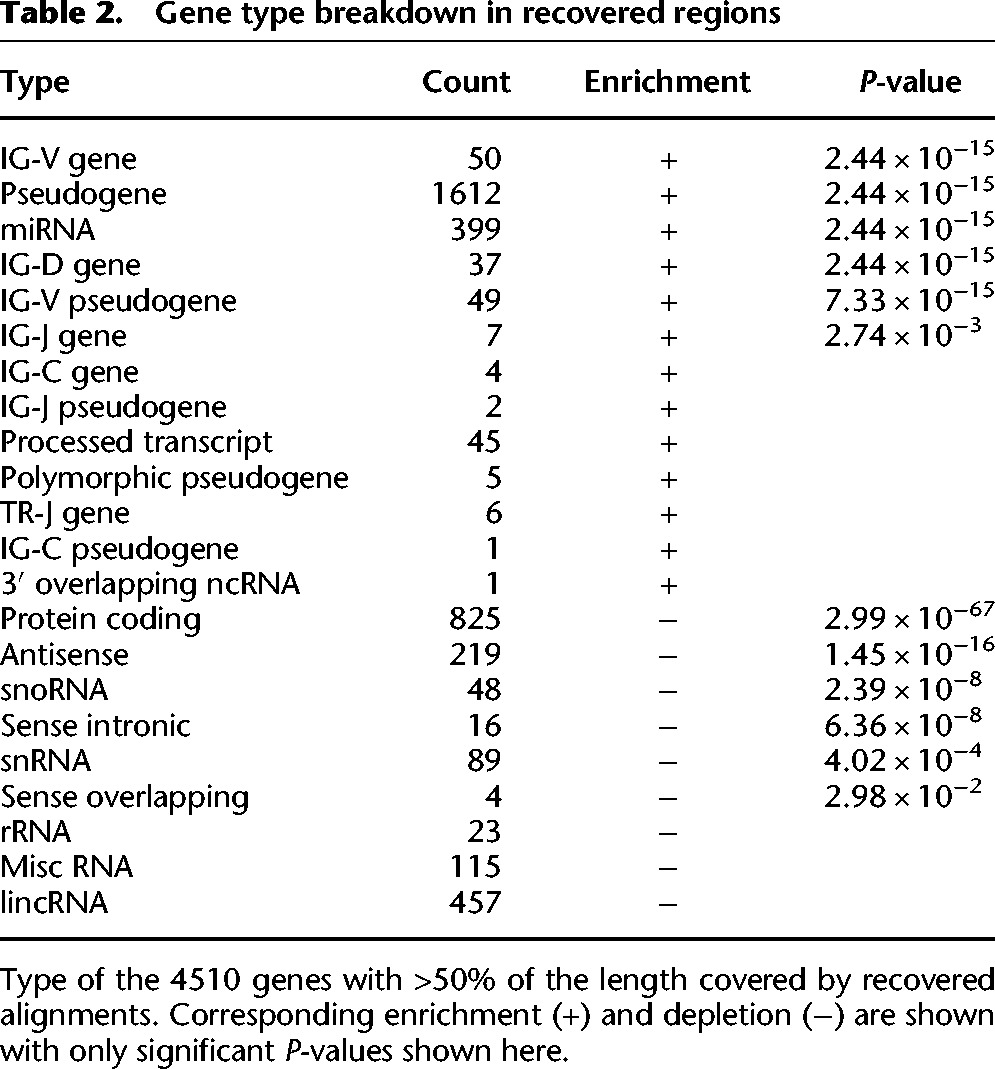
Gene type breakdown in recovered regions

**Table 3.**
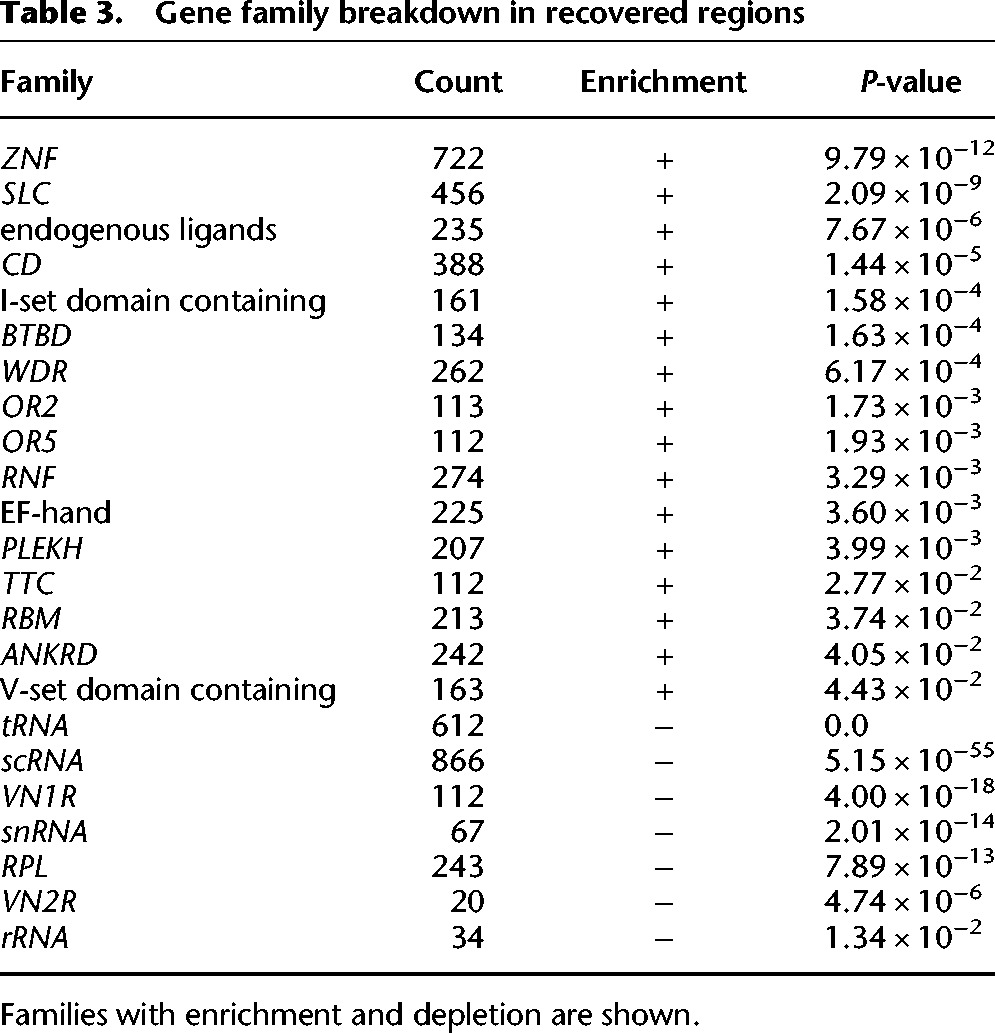
Gene family breakdown in recovered regions

### SNV identification within recovered regions

We next applied RFA to two shallow-sequenced TruSeq read cloud samples (*C*_R_ = 1.5×). We identified SNVs with these new alignments and performed validation in order to determine our variant discovery accuracy within repeat regions. For each sample, we aligned each well separately using RFA, applied the GATK (DePristo et al. 2011) pipeline to the resulting alignments of all wells simultaneously to recover candidate germline variants, and used computed queries to further filter these candidates to produce variants (see Supplemental Material for details). We compared the resulting calls with variant calls performed using the same GATK pipeline without application of RFA or read cloud information.

#### NA12878 sample

We first applied RFA to three shallow TruSeq read cloud libraries for the HapMap sample NA12878. The first library (*C*_*R*_ = 1.5×, *C*_*F*_ = 6.2×) (Kuleshov et al. 2014) had sufficiently different coverage properties from the other two (*C*_*R*_ = 0.5×, *C*_F_ = 12.5× for each lane), which were provided to us much later after the protocol had been extensively optimized for whole-genome haplotyping. Each well of the more recent libraries also contained a significantly higher fraction of the genome than the original library (3.4% versus 1.6%).

By applying RFA to these three lanes, we recovered an additional 50,314 variants (35,092 heterozygous) over those found using baseline short-read alignments within the 171 Mb of highly repeated sequences. We validated these recovered variants against three different sources of long-read sequencing data available for NA12878 (Table 4; see Supplemental Material for validation details):

1. A separate whole-genome sample that had been sequenced with high coverage assembled Illumina TruSeq synthetic long reads (*C*_*R*_ = 50×, *C*_*F*_ = 29×; Genomes Moleculo NA12878, 2014, op. cit.), which we aligned to the reference using BWA-MEM (Li 2013). The long reads provided sufficient coverage to directly validate 33,984 of our recovered SNV calls, with a validation rate of 99.5% for our homozygous calls and 92.0% for our heterozygous calls.
2. A set of BACs targeting large high-identity duplications in 15q13.3 that had been sequenced with PacBio (Antonacci et al. 2014). We used BWA-MEM to align the assembled BACs to the reference, and validated 301 of our recovered SNV calls overlapping the aligned regions. Of the variants that we identified within these regions, 97.6% of our homozygous calls and 53.1% of our heterozygous calls were validated. A validation rate of roughly ∼50% for heterozygous calls is expected since these clones are known to originate from a single haplotype in these regions.
3. A set of 103 fosmids that had been randomly selected across the whole genome (Weisenfeld et al. 2014) and sequenced with PacBio. We used BWA-MEM to align the assembled fosmids to the reference and validated 127 variants. Of the variants that we identified within the regions overlapped by these fosmids, 100% of our homozygous calls and 80% of our heterozygous calls were validated.

**Table 4.**
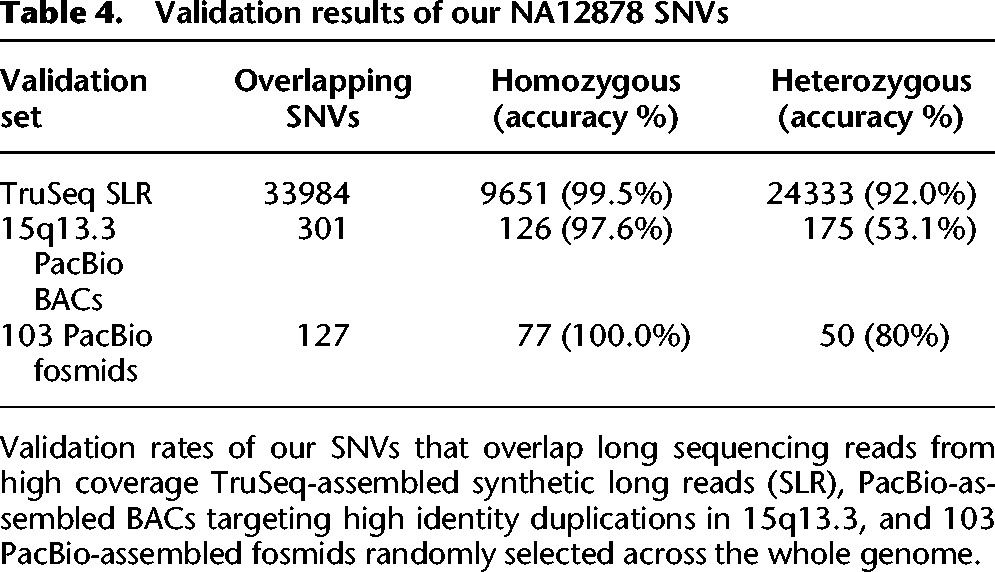
Validation results of our NA12878 SNVs

#### Invasive ductal carcinoma sample

We then applied RFA to high coverage sequence data obtained from a fresh frozen sample of a grade 3, *ERBB2* amplified, estrogen receptor (ER) negative invasive ductal carcinoma (IDC). Eight Moleculo libraries of total long fragment coverage *C*_*F*_ = 44× were sequenced at *C*_*R*_ = 1.8× to produce read clouds with a total short-read coverage of *C* = 78.5×. The high sequence coverage in this IDC sample allowed us to discover a total of 3,286,470 SNVs. Of these variants, 197,529 (6%) were found in the RFA alignments only and not in the baseline, representing a significant, previously hidden fraction of variation. We randomly selected a subset of the RFA-only variants within the 171 Mb of inaccessible sequence that was amenable to multiplex PCR validation. Of 346 submitted, 323 (93.4%) were validated as displaying an alternate allele frequency (AAF) of at least 0.1 (see Supplemental Material for validation details). Although these validated variants required at least one unique primer (up to one mismatch), and therefore are in principle biased to be within repeat regions with at least one unique nucleotide in range of the amplicon that distinguishes the copies, they provide additional support that matches our variant discovery accuracy measured in NA12878.

**Figure 4.**
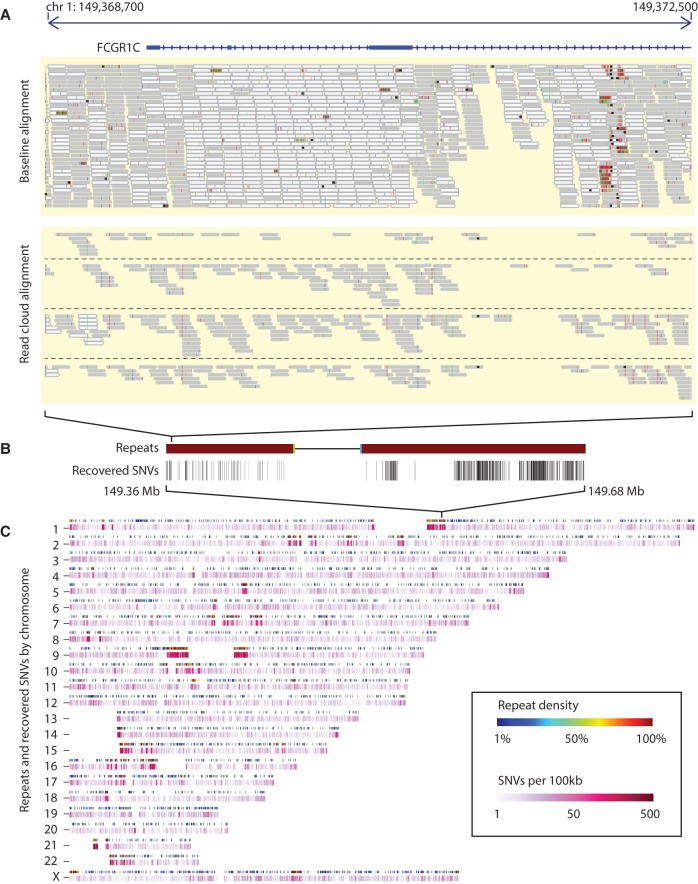
Whole-genome SNV calling on the IDC sample. (*A*) Comparison of the initial baseline short-read alignments of all the wells merged together with four wells aligned with RFA (from two distinct haplotypes), in a region overlapping the *FCGR1C* gene. (*B*) Placement of recovered SNVs within the surrounding 300-kbp region. (*C*) Density of recovered SNVs throughout the whole genome (*bottom* track), by chromosome, compared to density of segmental duplications (*top* track). Long clustered regions of recovered SNVs coincide with dense regions of annotated segmental duplications.

We determined the placement of our discovered variants in the IDC sample within each annotation category across the whole genome (Fig. 4). A comparison of the placement of our SNVs in the IDC sample against known, high sequence-identity segmental duplications indicates that a significant portion lie within dense stretches of recovered low copy repeats. We further examined the placement of the identified SNVs across all repeated sequence classes and found that 116,173 (59%) lie within segmental duplications and 16,521 (8%) lie within gene regions, with the rest residing within lower complexity elements (Table 5). We examined both the set of variants that were filtered (by the GATK pipeline) from the baseline run and recovered in the RFA run, as well as the set of variants which were unique to just the RFA run. The majority of variants (55%) were unique to RFA and showed no significant signal in the original baseline short-read alignments.

**Table 5.**
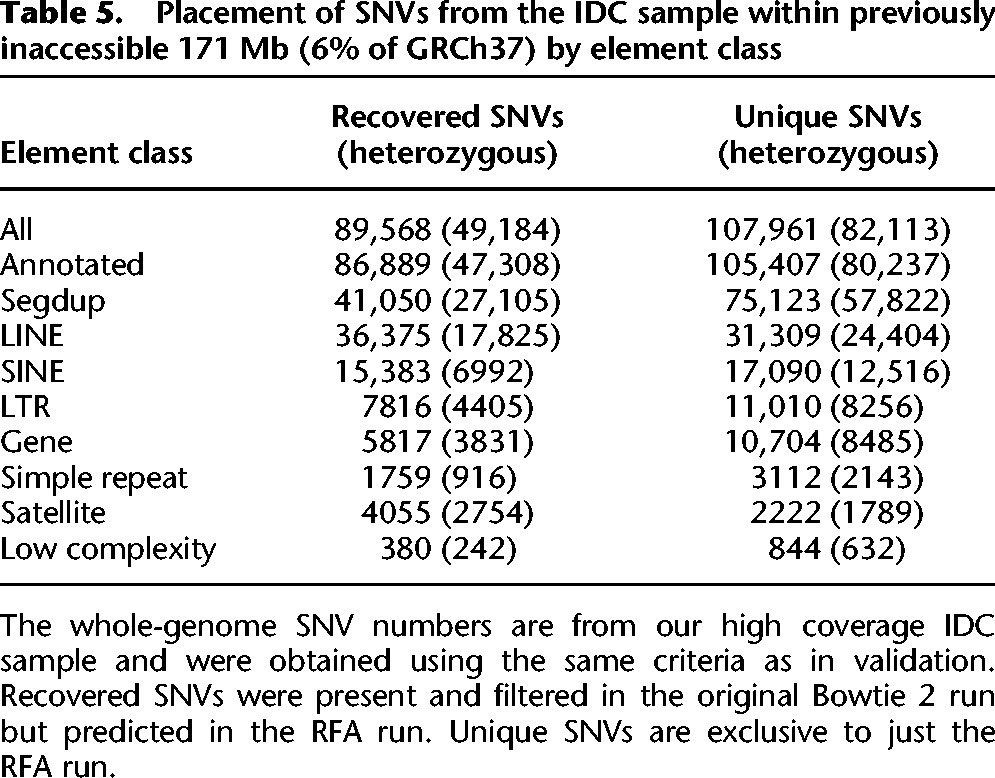
Placement of SNVs from the IDC sample within previously inaccessible 171 Mb (6% of GRCh37) by element class

RFA is able to confidently align reads to 171 Mb of the human genome previously inaccessible to short-read technologies. Validation with assembled synthetic long reads, PacBio assembled clones, as well as PCR and deep targeted sequencing confirm that these alignments can be used for accurate nucleotide-level variant discovery. Examination of both alignments and SNVs in the IDC sample indicates that RFA enables variant discovery across all classes of repeated sequence, including genes, high copy repetitive elements, and high sequence identity segmental duplications.

## Discussion

In this work, we demonstrate the ability of RFA to leverage read clouds to accurately map short reads in 171 Mb of the human genome that is inaccessible to variant calling using short reads. Our method enables the use of the TruSeq synthetic long-read protocol in a cost-effective, read cloud setting, in which each long molecule is covered lightly with reads (*C*_*R*_ ≤ 2×) rather than requiring long molecules to be assembled into synthetic long reads through deep sequencing (*C*_*R*_ = 50×). Using a probabilistic approach, we jointly map all reads of a well to the reference genome to produce confident unique mappings that subsequently enable accurate discovery of novel nucleotide variation in this previously dark portion of the human genome.

Our approach models the features of a given synthetic long-read protocol in *P*(***M***). For TruSeq read clouds, we chose to decompose and learn this function with empirical distributions, but replacing it would allow our approach to be readily adapted to other synthetic long read technologies.

Although we provide proof of concept of our method for discovering copy-specific SNVs on a whole-genome human sample, there are numerous additional potential applications of aligning read clouds. In future work, our improved alignments can be leveraged to discover other types of variation, including indels and larger structural rearrangements present in these regions. Our preliminary analyses of the recovered alignments in the human genome also suggest a copy-rich structure that has yet to be resolved accurately beyond illuminating SNV variation.

Our approach can be used to resolve ambiguity in complex regions of high sequence identity. It could be useful in resolving ambiguity in homologous regions, such as MCS and LRC, for which the updated GRCh38 human reference genome contains multiple alternative haplotype paths (Church et al. 2011). Aligning with RFA may enable determination of the correct haplotype paths of these variable regions. Our approach may also prove useful for subspecies discovery and quantification in metagenomic samples for which previous work had much higher sequencing requirements using TruSeq-assembled synthetic long reads (Sharon et al. 2015). There has been previous work to leverage PacBio sequencing to accurately resolve RNA transcripts (Sharon et al. 2013). With some modeling extensions, RFA can potentially be used to align directly to an available transcriptome (of which many transcripts will have high sequence identity) or for discovery of novel transcripts with previously unknown alternative splicing sites.

Our current method has several limitations. First, alignment generation with standard short-read aligners limits our method's ability to fully illuminate high copy-number elements because they may not generate any concordant paired alignments for reads originating from high-copy regions. If the true candidate alignment for a particular read is not produced by short-read alignment during pass2, then our method will be unable to find the true mapping for that read. However, our approach naturally lowers the quality scores for alignments in high-copy regions such that they do not affect further analyses. Second, although our inference procedure performed well in practice, it may benefit from an improved proposal distribution to explore the state space of possible alignment configurations more effectively and further close the gap with respect to Oracle performance. Finally, breaking the independence assumption to allow variant information to be shared across wells may improve variant discovery accuracy and recall.

Of the 130.5 Mb that are identified as high sequence identity segmental duplications (Bailey et al. 2002), a significant portion is currently hidden from short-read technologies, and the effect of variation in these regions is largely unknown. High-identity segmental duplications are highly variable between individuals, and copy number variants (CNVs) within these regions are strongly correlated with increased sequence identity (Redon et al. 2006). Nonallelic homologous recombination between LCRs as well as *Alu* repetitive elements can result in a variety of balanced and unbalanced structural alteration events (Redon et al. 2006; Sasaki et al. 2010; Ou et al. 2011). These regions are enriched for transcript content, and a subset of these regions consists of multicopy genes also known to vary widely in copy number between populations and individuals (Sudmant et al. 2010). Illumination of these highly dynamic regions will enable discovery of variation across individuals, and ultimately, functional annotation and phenotype association.

## Methods

In order to leverage read clouds for accurate read alignment, we model the long read generative process to capture the dependencies between the resulting short reads through the hidden long fragments. Using a standard short-read aligner to provide seeded candidate long molecules and short-read alignments, we are then able to reduce the problem of finding optimal alignments to optimizing over an MRF. We first define our model and our objective function and then show how we can use this framework to obtain alignments and their respective mapping quality scores.

### Definitions

For wells ***W*** = {1,2, … ,*W*}, we set up the following system to describe the long read process. Each *w* ∈ ***W*** contains a set of read *fragments* ***R***_*w*_ = {*R*_*wn*_|*n* = 1,2, … ,*N*_*w*_}. Each read fragment, *R*_*wn*_, can potentially have multiple alignments, and we denote ***A***_*wn*_ = {*a*_*wnk*_|*k* = 1,2, … ,*K*_*wn*_} as the set of its possible alignments with respect to the reference genome. We incorporate our knowledge of the synthetic long-read process by modeling the set of original long molecules ***M***_*w*_ = {*M*_*wi*_|*i* = 1,2, … ,*I*_*w*_} that generated the short reads.

For each well *w*, the process can be described by the following (*w* is omitted for clarity):

1. A set of hidden random vectors denoting long molecules that generated the short reads:
  $$M=\{M_{c}|c=1,2,\ldots ,C\}.$$

To describe *M*_*c*_, we feature it as (*L*_*c*_, ***X*_*c*_**, λ_*c*_, *B*_*c*_, *S*_*c*_, *E*_*c*_). *L*_*c*_ is the aligned position in the reference; ***X*_*c*_** represents the hidden sequence of the molecule; and *S*_*c*_ is the size of the molecule. λ_*c*_ controls the short-read emission random process. *B*_c_ captures the presence of the end-markers and can be stored as a conditional probability table (CPT). *B*_*c*_, *S*_*c*_, and λ_*c*_ can all interact depending on the particular long-read protocol, so we model them jointly as *P*(*B*_*c*_, *S*_*c*_, λ_*c*_). For modeling convenience, *E*_*c*_ denotes whether this source molecule existed in this well, and *E*_c_ ∼ Ber(*p*^*e*^).

2. A set of partially observed random vectors denoting read fragments:
  $$R=\{R_{n}|n=1,2,\ldots ,N\}.$$

We represent *R*_*n*_ as (***o***_*n*_, *A*_*n*_) where ***o***_*n*_ is the observed information of the read such as nucleotide sequence and base quality scores, and *A*_n_ is the hidden alignment and captures the differences between the reference and the read for this particular alignment.

The distribution over the read fragments, over which we seek to optimize, can be described by

(2) $$P(R)=\;\underset{M}{\sum }P(M,R),$$

where the sum is over all possible hidden long fragment configurations.

### Long-read alignment seeding

**Figure 5.**
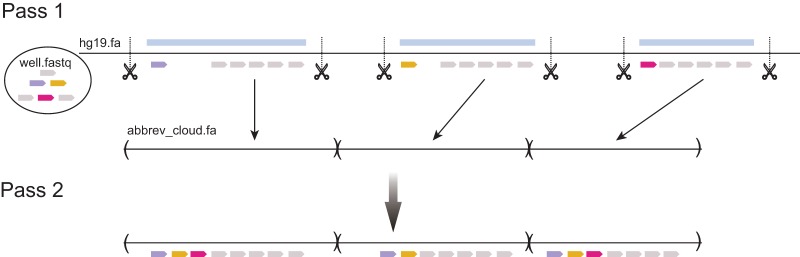
Abbreviated reference framework. The framework for generating putative long reads and associated short-read alignments to these segments: (1) Reads are aligned to hg19 at most once in pass1; (2) putative long read segments are identified and spliced together to create an abbreviated reference; and (3) reads are aligned again in pass2 to this abbreviated reference allowing multiple mappings.

Although the original long molecules themselves are completely latent, we can predetermine a set of partially observed candidate long molecules

$$\left\{M_{c}=(L_{c}=l_{c},X_{c},\lambda_{c},B_{c},S_{c}=s_{c},E_{c})|c=1,2,\ldots ,C\right\}$$

as follows (Fig. 5). Reads are aligned to the full hg19 reference (*pass1*) using a short-read aligner allowing at most one alignment per read. We pass through the resulting alignments and use a heuristic set of rules to detect candidate clouds corresponding to clusters of reads. We group reads that are within 3.5 kbp of one another into the same cluster and require a cluster to have a minimum of six reads to constitute a candidate cloud. We adopt a simple heuristic to leverage the TruSeq long-fragment end-marker information to split a candidate cloud in two in the cases in which two long fragments happen to be within 3.5 kbp of one another (without mixing overlap) and would otherwise be clustered by our simple rule above. The choice of 3.5 kbp as the distance between reads was made using training simulations to minimize the number of superfluous candidate clouds (not representing true sampled long fragments) while also minimizing false negative clouds so that true candidates are not missed. After candidate cloud calling, we then create an abbreviated reference containing a separate contig for each candidate long molecule. All reads are then realigned to this abbreviated reference (*pass2*) allowing multiple candidates per read.

The resulting alignments serve as seeded domains *A*_*n*_ for each read *R*_*n*_. Definition of the abbreviated reference helps in filtering spurious alignments to distant homolog sequences, which should not be considered as potential source molecules. In practice, we found that a lenient threshold for calling candidate clouds in pass1 was sufficient to retain nearly all the true candidate clouds containing ambiguous reads.

### Read graph construction

In order to perform inference, we make the following simplifying assumptions:

1. The underlying sample sequence in one well does not provide any information about the underlying sequence in other wells such that
  $$P(R)=\underset{w}{\prod }P(R_{w}).$$
2. Within each well, the long molecules do not overlap in the same genomic region in the reference genome.

The process to generate read clouds in one well (dilution, shear into short fragments, and barcoding) is unlikely to affect the observed reads in any other well. However, since the wells share the same underlying germline genome, the observed short reads in each well are actually not independent and interact through the unobserved underlying sample sequence. Although our first assumption in principle limits the ability of our model to leverage all available information, it is a significant simplification that allows us to efficiently perform alignment on each well separately. Regarding the second assumption, there is actually a nontrivial chance two fragments sample overlapping genomic coordinates in a single well, equal to the well genome coverage, which was ∼2% in our samples. However, the end-markers from TruSeq allow us to detect most of these collisions when they do occur. We estimated the end-marker efficiency for our samples to be *p* = 0.77; assuming independence of the end-markers of two overlapping fragments allows us to detect 1 − *p*^2^ = 95% of collisions. There is an additional, subtle assumption here: In order for fragments to be nonoverlapping from one another, they would have to be sampled from a nonuniform distribution such as the “parking process” (Krapivsky 1992). However, when the total fragment length is small compared to the total target genome length, the uniform distribution of sampled fragments (Lander and Waterman 1988) is a good approximation of the parking process (Batzoglou et al. 1999). All together, we approximate the long fragments as being nonoverlapping, but otherwise independently drawn, and decompose read assignment scores by candidate long molecules for tractable computation

$$P(M_{w})\approx \underset{c}{\prod }P(M_{wc}).$$

The assumptions above allow us to decompose the global probabilistic function in Equation 2 to

(3) $$P(R)=\underset{c}{\prod }\underset{M_{c}}{\sum }P(M_{c})P(R_{c}|M_{c}),$$

where *c* indexes over clouds, and *R*_*n*_ ∈ ***R***_c_ if there is at least one alignment in *A*_n_ within the coordinate range [*l*_*c*_,*l*_*c*_ + *s*_*c*_). The sum over *M*_*c*_ is over all possible hidden values of the domain of the features defined in the previous section. This results in a Markov Random Field (MRF) with each long molecule inducing a potential function φ_c_(***R***_*c*_) of the form above $\left[\varphi_{c}(R_{c})=\underset{M_{c}}{\sum }P(M_{c})P(R_{c}|M_{c})\right]$ such that the distribution of read assignments can be represented as

$$P(R)=\frac{1}{Z}\underset{c}{\prod }\varphi_{c}(R_{c}).$$

For a particular read assignment, ***R***_*c*_ = (***o***_*c*_, ***a***_*c*_), *L*_*c*_, *B*_*c*_, *S*_*c*_, and *E*_*c*_ can be trivially inferred to result in only one significant value in each of their domains (shown as constants below). To compute the desired potential value, *P*(***R***_*c*_), we still have to integrate or sum over the domain of λ_*c*_ and ***X*_*c*_**. Our prior of long molecule features decomposes as

(4) $$\begin{array}{rl} P(M_{c}) & =P(L_{c}=l_{c},X_{c},\lambda_{c},B_{c},S_{c}=s_{c},E_{c}=e_{c})\\ & =P(E_{c}=e_{c})P(B_{c}=b_{c},S_{c}=s_{c},\lambda_{c}|E_{c})P(L_{c}=l_{c}|E_{c})\prod_{X_{l}\in X_{c}}P(X_{c}|E_{c}) \end{array}$$

*E*_c_ is a binary random variable, which is 1 if any reads are assigned to *M*_c_ and 0 otherwise. Note that *P*(*L*_*c*_, *B*_*c*_, *S*_*c*_, *λ*_*c*_, ***X*_*c*_**|*E*_*c*_ = 0) = 1. *P*(*X*_l_|*E*_c_ = 1) ∼ Cat(θ^*l*^) captures our belief of the SNP mutation rate at position *l* in the reference genome (due to germline plus somatic variation). In our implementation, we estimated a shared parameter by determining the germline SNP rate at unique regions in the genome. We chose *P*(*L*_*c*_|*E*_*c*_ = 1) to be uniform, but the bias of a particular protocol to sample certain long fragments over others could be captured in this function. Decomposition of the prior as in Equation 4 allows us to compute the potential φ_*c*_(***R***_*c*_) efficiently in linear time by variable elimination of ***X*_*c*_** and λ_*c*_.

Our approximate inference procedure relied on the ability to compute the score of an updated assignment efficiently using the decomposition across candidate long molecules in Equation 3 and is explained in the Supplemental Section MAP Inference Procedure. Although we obtained good results by decoupling the wells, this assumption can be loosened to allow interaction between wells in order to share information and improve performance. Each well has information about the set of underlying variants ***X***, but contains a different set of observations through its subset of reads. Allowing wells to share their belief about ***X*** would affect the joint alignment and in principle may result in improved performance. Although our assumption that long fragments in each well do not overlap in genomic coordinates was useful in deriving our model, it is often not true in practice. Fortunately, the presence of the end-marker short reads in TruSeq sequencing allowed us to detect most of the cases when sampled long fragments overlapped. The respective reads were excluded from our analysis since we do not have the ability to tell from which long fragment the short reads originated in the case of an overlap.

### Query computation

#### Read quality scores

We compute maximum a posteriori (MAP) read alignments (see Supplemental Material for details) and infer quality scores for the alignments as follows. We assume that once the MAP assignment converges, reads only have local interactions such that

$$P(R_{n})=P(R_{n}|R_{l-}=r_{l-}^{\mathrm{MAP}}),$$

where ***R***_*l*−_ denotes the set of reads which do not overlap *R*_*n*_ in the identified MAP assignment. We can then compute

$$\begin{array}{rl} P(R_{n}|R_{l-}) & =\;\underset{R_{l-n}}{\sum }P(R_{l}|R_{l-})\\ & =\underset{R_{l-n}}{\sum }\underset{X}{\sum }P(R_{l},X|R_{l-})\\ & =\underset{R_{l-n}}{\sum }\underset{X}{\sum }P(R_{l}|X,R_{l-})P(X|R_{l-}), \end{array}$$

where ***R***_*l−n*_ denotes ***R***_*l*_ excluding *R*_*n*_; and ***X*** is the union of sequence at all the potential locations ***R***_*l*_ could be mapped to. The small sizes of both ***R***_*l−n*_ and ***X*** allow this quantity to be computed efficiently. The computed *P*(*R*_*n*_|***R***_*l*__−_) can then be converted to a MAPQ score for that read.

With our method, alignments converge in the MAP assignment such that certain candidate long molecules are empty, and the corresponding candidate alignments within these coordinates can be eliminated from the domains ***A***_*n*_. If the resulting domains have only one alignment active, as is often the case for low copy repeats, then the quality score will be high. However, the quality score for a recovered alignment will be lowered if it falls in elements that are repeated with high sequence identity within the resulting active long molecules, in which cases *P*(*R*_*n*_|***R***_*l*__−_) will be naturally lowered.

#### Cloud quality scores

For each cloud, we can directly compute $P(R_{c})=\underset{M_{c}}{\sum }P(M_{c})P(R_{c}|M_{c})$ for the identified MAP assignment by variable elimination as described in the previous section. This quantity in log-space is directly proportional to the number of variants predicted in a long fragment for a given assignment, ***R***_*c*_. Low-quality long molecules may correspond to novel copy number variants in the sample that are not present in the reference and whose sequence identity with the reference homolog where they map is unusually low. We exclude short reads within clouds with low quality scores from our variant calling pipeline. A pooling strategy that collects all such read clouds to assemble additional potential copy number variants in the target genome is an interesting subject for future work.

Our read quality score, $P(R_{n}|R_{l-}=r_{l-}^{\mathrm{MAP}})$, does not capture cases in which the alignment may be unique within an active long molecule, but all the reads of that molecule could map nearly equally well to an inactive long molecule. We approximated $P(E_{c}|R_{c-}=r_{c-}^{\mathrm{MAP}})$ (which cannot be computed exactly due to the size of ***R***_*c*_), and chose an appropriate cutoff using training simulations for excluding alignments in order to minimize error. We can approximate it effectively by identifying the set of assignments to inactive clouds $\left\{r_{c_{1}},\ldots ,r_{c_{k}}\right\}$, computing *P*(***X***_*c*_|***r***_*c*_) for each one, and then renormalizing. This is an inexpensive way to identify the peaks of the distribution since upon convergence, the assumption is that interactions between reads of different long molecules are mostly decoupled. The result can be used to exclude long molecules for which all the member read fragments can map equally well in another source molecule, *c*′, thus making them indistinguishable.

#### Simulations of read clouds

We used a nonparametric approach in order to simulate read clouds and capture biases intrinsic to the sequencing process (see Supplemental Fig. 1). We first aligned all the wells from our IDC sample and generated candidate clouds using the same criteria described in pass1. We removed outlier clouds in regions with low mappability (corresponding to repeats) or where we observed unusually high coverage likely due to copy number variants. The remaining set of read clouds was assumed to be representative of true sequenced read clouds that are present across the whole genome. For each read cloud in this set, we created a stencil of the short-read positions relative to the start of the cloud to be used as a “cookie cutter” for generating short reads in a simulated read cloud. In order to simulate base substitution errors in reads, we fit a first-order model of the read bases and base quality scores from our sequenced sample. We introduced a substitution error with probability proportional to the simulated base quality score. To simulate reads from a well, we choose a location uniformly at random in the reference and draw a stencil that has not yet been used in order to create a sampled long fragment.

We repeat the process until the well contains the expected genome coverage (2% in our samples). The use of this empirical simulation methodology, rather than use of a model-based simulation, enables us to more accurately capture intrinsic biases present in read-cloud sequencing and to better estimate the true accuracy of our alignment approach. Still, we recognize that this simulation strategy does not capture any sequence-specific biases of the read-cloud and sequencing protocols.

### TruSeq instantiation

To align short reads to the reference, we chose to use Bowtie 2 (Langmead and Salzberg 2012) for its high efficiency and accuracy and for its ability to output multiple candidate alignments for each read. We restricted the number of alignments for each read to be 15. Reads with possibly more alignments were unlikely to be informative in the placement of other reads.

We found that the distributions of *B*_*c*_, *S*_*c*_, and λ_*c*_ for TruSeq read clouds were difficult to parameterize. The read-cloud size and density varied greatly conditioned on the presence of the end markers, so we factorized *P*(*B*_*c*_,*S*_*c*_,λ_*c*_) as *P*(*B*_*c*_)*P*(*S*_*c*_,λ_*c*_|*B*_*c*_). These functions were estimated with Kernel Density Estimates (KDEs) on features extracted from the valid clouds described in the simulations section.

### Implementation and availability

RFA is a Python package that leverages the short-read aligner Bowtie 2. It is open source and freely available at http://readclouds.stanford.edu and in the Supplemental Material. To align a single lane of sequenced read clouds, a subset of the wells are aligned using the default Bowtie 2 settings, and features of uniquely mapped clouds are extracted to learn the cloud model *P*(***M***). To achieve practical runtimes on large genomes, our implementation required the use of a compute cluster to parallelize alignment across wells. Each well is aligned in the following main steps: (1) Align all reads to the whole reference once; (2) determine abbreviated reference and align all reads to this abbreviated reference allowing multiple candidates; (3) build in memory structures and the read MRF graph; (4) perform MAP inference to determine optimal alignments; and (5) compute alignment quality scores and generate the final alignment files. For the first lane of NA12878 sequencing (*C*_*R*_ = 1.5×, *C*_*F*_ = 6.2×), for which we initially designed our implementation, steps 1–5 took 40, 76, 57, 29, and 49 CPU hours, respectively, across all 384 wells. Our method requires about 6× more total CPU time than generating baseline alignments (step 1). In future software releases, steps 1–3 may be folded into a single step and further optimized, and steps 3–4 may be implemented in a compiled language.

## Data access

All raw sequence reads for the eight Illumina TruSeq libraries for the IDC sample as well as the three libraries for NA12878 have been submitted to NCBI BioProject (http://www.ncbi.nlm.nih.gov/bioproject/) under accession number PRJNA287848.
